# Psychometric properties of the Patient Health Questionnaire-9 and the Generalized Anxiety Disorder-7 in Mexican cancer patients

**DOI:** 10.1017/S1478951526102752

**Published:** 2026-06-08

**Authors:** Oscar Galindo-Vázquez, Erika Ruíz-García, Rosa María Álvarez-Gómez, Abel Lerma Talamantes, Ana Marcela González Ling, Mónica Ramírez Orozco, Ramiro Espinoza Zamora, Alejandra Berenice Uriostegui Garduño, Javier Eliud Núñez-Hernández, Marcos Espinoza Bello, Rosario Costas-Muñiz

**Affiliations:** 1 Psycho-Oncology Service, National Cancer Institute (INCan), Mexico City, Mexico; 2 Chief Academic Office / Translational Medicine Laboratory, National Cancer Institute (INCan), Mexico City, Mexico; 3 Hereditary Cancer Clinic, National Cancer Institute (INCan), Mexico City, Mexico; 4 Institute of Health Sciences (ICSa), Autonomous University of the State of Hidalgo (UAEH), Pachuca, Mexico; 5 Department of Breast Cancer, National Cancer Institute (INCan), Mexico City, Mexico; 6 Department of Hematology, National Cancer Institute (INCan), Mexico City, Mexico; 7 Full-time Professor, Bachelor of Nutrition Program, Faculty of Higher Studies Zaragoza (FES Zaragoza), National Autonomous University of Mexico (UNAM), Mexico City, Mexico; 8 Department of Psychiatry & Behavioral Sciences, Memorial Sloan-Kettering Cancer Center, Immigrant Health & Cancer Disparities Service, New York, NY, USA

**Keywords:** Anxiety, cancer, depression, psychometric properties, Mexico

## Abstract

**Objectives:**

To examine the psychometric properties of the Patient Health Questionnaire-9 (PHQ-9) and the Generalized Anxiety Disorder-7 (GAD-7) in Mexican cancer patients, and to evaluate their utility as brief screening measures for depressive and anxiety symptoms in oncology care.

**Methods:**

In this cross-sectional study, 357 adult patients receiving oncological treatment at a cancer hospital in Mexico completed the culturally adapted Spanish versions of the PHQ-9 and GAD-7. Exploratory and confirmatory factor analyses were conducted to assess the factorial structure of both instruments, and internal consistency was evaluated using Cronbach’s alpha. Concurrent validity was examined through correlations with related measures.

**Results:**

The PHQ-9 yielded a 2-factor structure with acceptable internal consistency (Cronbach’s *α* = 0.837), while the GAD-7 showed a 1-factor structure with good internal consistency (Cronbach’s *α* = 0.881). The PHQ-9 explained 55.3% of the variance and the GAD-7 explained 58.5%. Confirmatory factor analyses indicated adequate model fit for both instruments.

**Significance of results:**

Findings suggest that the PHQ-9 and GAD-7 are brief, valid, and reliable tools for detecting depressive and anxiety symptoms in Mexican cancer patients. Their use may facilitate the early identification of symptoms of anxiety and depression in oncology settings and support both clinical care and psycho-oncology research.

## Introduction

Anxiety and depressive symptoms are among the most prevalent mental health concerns in patients with cancer and are consistently associated with poorer quality of life, greater symptom burden, reduced treatment adherence, and increased healthcare utilization (Pelletier et al. 2002; Galindo Vázquez et al. 2015). Meta-analytic and large-scale studies indicate that approximately 1 in 4 patients experience clinically significant depressive symptoms and nearly 1 in 3 report anxiety, yet a substantial proportion do not receive psychological support, particularly those with metastatic disease, female-specific cancers, or limited partner support (Brintzenhofe-Szoc et al. 2009; Linden et al. 2012; Shalata et al. 2024; Getie et al. 2025). This treatment gap is further underscored by recent evidence showing that nearly 40% of patients perceive a deterioration in their mental health, while only about half receive psychological care (Shalata et al. 2024).

Depression in oncology has been linked to increased physical symptomatology, diminished survival, and elevated mortality risk, with estimates suggesting a 23–83% increase in cancer-related mortality depending on tumor type and study design (Bortolato et al. 2017; Inchausti et al. 2020; Ungvari et al. 2025). Anxiety is similarly consequential, often intensifying at diagnosis and during disease progression, and may impair decision-making, treatment adherence, and communication with healthcare providers (Traeger et al. 2012; Bronner et al. 2018; Getie et al. 2025). Although some patients do not meet full diagnostic criteria for depressive or anxiety disorders, subthreshold symptoms can still significantly affect functioning and quality of life and may benefit from timely psychosocial intervention (Bail et al. 2018; Larkin 2020).

International clinical guidelines consistently recommend routine screening for depression and anxiety using brief, validated instruments such as the Patient Health Questionnaire (PHQ-9) and the Generalized Anxiety Disorder Scale (GAD-7) (Joffres et al. 2013; Cleare et al. 2015; Bandelow et al. 2022). The PHQ-9, developed by Kroenke et al., is based on DSM-IV criteria for major depressive disorders and has demonstrated strong psychometric properties and clinical utility across diverse populations (Kroenke et al. 2001). Similarly, the GAD-7, developed by Spitzer et al., is grounded in DSM-IV criteria for generalized anxiety disorder and has shown robust validity, reliability, and a predominantly unidimensional factorial structure in multiple validation studies(Spitzer et al. 2006; Orozco et al. 2013).

Although validated instruments exist in the Mexican population to assess anxiety and depressive symptoms (Galindo Vázquez et al. 2015; Almeida et al. 2023), they do not fully align with diagnostic criteria for major depressive disorder and generalized anxiety disorder, and evidence regarding their validity, reliability, and confirmatory factorial structure in Mexican cancer patients is limited. Furthermore, recent umbrella reviews highlight the substantial and persistent burden of anxiety and depression among cancer patients and survivors, including during the COVID-19 pandemic, reinforcing the need for systematic mental health screening in oncology (Getie et al. 2025). Therefore, this study aimed to evaluate the psychometric properties of the PHQ-9 and GAD-7 in a sample of Mexican patients with cancer. Establishing their validity and reliability in this population will support their integration into routine clinical practice and research, contributing to improved identification and management of psychological symptoms in oncology.

## Methods

### Study participants

This cross-sectional study included adult patients receiving oncological care at a cancer hospital in Mexico. Participants were recruited consecutively during outpatient medical consultations and provided written informed consent prior to enrollment. Inclusion criteria were: (a) confirmed cancer diagnosis of any type or stage, (b) current oncological treatment, and (c) ability to read and write. Exclusion criteria included: (a) acute psychological crisis as determined by the clinical interviewer and (b) severe cognitive, auditory, or visual impairments that could interfere with questionnaire completion. These criteria ensured adequate comprehension and minimized measurement bias.

### Ethical aspects

The study was reviewed and approved by the institutional Research and Ethics Committees, in accordance with applicable ethical standards (Approval Codes: 025/025/POI and CEI/012/25), in accordance with the Declaration of Helsinki. All participants provided informed consent after receiving detailed information about study procedures and confidentiality safeguards.

### Cultural adaptation

A cultural adaptation process was conducted following international guidelines for cross-cultural validation of psychological instruments (Koller et al. 2007). A pilot test was administered to 30 Mexican patients using the official Spanish versions of the PHQ-9 and GAD-7 (available at www.phqscreeners.com). Participants completed a structured cognitive debriefing form assessing clarity, comprehension, and potential cultural or linguistic issues in each item. Feedback was reviewed by the research team, and minor wording adjustments were made to enhance clarity while preserving conceptual equivalence. No items required removal or substantive modification.

### Measures

#### Data sheet

A standardized identification form collected sociodemographic data (age, sex, marital status, education, residence) and clinical information (cancer type, stage, treatment modality, and time since diagnosis).

#### Patient Health Questionnaire (PHQ-9)

The PHQ-9 is a 9-item screening instrument based on DSM-IV criteria for major depressive disorder (Kroenke et al. 2001). Items are rated on a 4-point Likert scale (0 = not at all to 3 = nearly every day), assessing symptoms over the previous 2 weeks. Total scores range from 0 to 27, with established cutoffs indicating mild (5), moderate (10), moderately severe (15), and severe depression (20). A score ≥ 10 is commonly used to identify clinically significant depressive symptoms. The PHQ-9 has demonstrated strong internal consistency and a predominantly unidimensional structure across populations (Kroenke et al. 2001), though 2-factor solutions have been reported in medical samples (Richardson and Richards 2008; Elhai et al. 2012; Petersen et al. 2015; Hinz et al. 2016).

#### Generalized Anxiety Disorder Scale (GAD-7)

The GAD-7 is a 7-item measure assessing anxiety symptoms based on DSM-IV criteria for generalized anxiety disorder (Spitzer et al. 2006). Items are rated on the same 0–3 Likert scale as the PHQ-9. Total scores range from 0 to 21, with cutoffs of 5, 10, and 15 indicating mild, moderate, and severe anxiety, respectively. A score ≥10 is typically used to identify clinically significant anxiety. The GAD-7 has consistently shown a unidimensional structure and excellent internal consistency across diverse populations (Spitzer et al. 2006; Orozco et al. 2013).

### Statistical analysis

#### Exploratory factor analysis

Descriptive statistics were computed for all items. Item discrimination was evaluated using independent-samples *t*-tests comparing the 25th and 75th percentile groups. Items with *p* > 0.05 were excluded from subsequent analyses. Inter-item correlations were examined, and sampling adequacy was assessed using the Kaiser–Meyer–Olkin (KMO) index and Bartlett’s test of sphericity. EFA was conducted using varimax rotation, retaining items with factor loadings ≥0.40 and eigenvalues >1. Internal consistency was evaluated using Cronbach’s alpha.

#### Confirmatory factor analysis

A confirmatory factor analysis (CFA) was conducted to evaluate the factorial structure identified in the exploratory factor analysis (EFA). Models were estimated using maximum likelihood estimation. Model fit was assessed using multiple indices: chi-square (χ^2^), chi-square/degrees of freedom ratio (χ^2^/df), Goodness-of-Fit Index (GFI), Adjusted GFI (AGFI), Comparative Fit Index (CFI), Tucker–Lewis Index (TLI), Root Mean Square Error of Approximation (RMSEA), and Hoelter’s critical N. Modification indices were examined to identify potential improvements while maintaining theoretical coherence. Analyses were conducted using AMOS version 23.

## Results

A total of 357 patients participated in the study. The mean age was 56 years, and 59% were women. Most participants were married (46%) and resided in Mexico City (37%). The sample included patients with advanced disease, with 34.5% diagnosed at stage III and 32.0% at stage IV. Breast cancer was the most frequent diagnosis, consistent with national epidemiological patterns (24). Sociodemographic and clinical characteristics are presented in Table 1.

**Table 1.** Sociodemographic and clinical characteristics of 357 cancer patients Table 1 long description.

Variable	Data	Variable	Data
Sociodemographic characteristics			
**Age** (years)	56 (44–65)		
**Sex**		**Marital status**	
Female	211 (59%)	Single	96 (26%)
Male	146 (41%)	Married	165 (46%)
**Residence**		Divorced	49 (13%)
Mexico City	131 (37%)	Widow	20 (5%)
Other states	226 (63%)	Other	27 (7%)
**With children**			
Yes	274 (77%)		
No	83 (23%)		
**Number of children**	2 (1–3)		
Clinical characteristics			
**Cancer stage**		**Comorbidities**	
I	75 (21%)	Yes	118 (33%)
II	90 (25%)	No	239 (67%)
III	76 (22%)		
IV	113 (32%)		
**Cancer type**		**Type of comorbidity**	
Breast	120 (34%)	Hypertension	35 (10%)
Prostate	43 (12%)	Diabetes	34 (10%)
Head & neck	33 (9%)		
Testicle	33 (9%)	Hypertension and diabetes	14 (4%)
Kidney	19 (5%)	Other	36 (10%)
Ovary	18 (5%)	**Karnofsky**	90 (90–100)
Bladder	17 (5%)		
Melanoma	12 (3%)	**ECOG**	1 (0–1)
Sarcoma	12 (3%)		
Other	50 (14%)		

Data are presented as absolute frequencies and percentages, as well as the median and the 25th and 75th percentiles.

The PHQ-9 demonstrated adequate internal consistency (Cronbach’s *α* = 0.837). Sampling adequacy was confirmed by a KMO value of 0.871 and a significant Bartlett’s test (*p* < 0.001). Inter-item correlations ranged from low to moderate. EFA using varimax rotation yielded a 2-factor solution with eigenvalues >1, explaining 55.3% of the total variance. Factor 1 included somatic and affective symptoms, while Factor 2 captured cognitive symptoms such as feelings of failure and thoughts of death, consistent with previous findings in cancer and chronic illness populations (Richardson and Richards 2008; Elhai et al. 2012; Petersen et al. 2015; Hinz et al. 2016). Item-level descriptive statistics and factor loadings are shown in Table 2.

**Table 2.** PHQ-9 factor analysis in cancer patients^a^ Table 2 long description.

Items	Factor 1	Factor 2	Mean	SD
4. Feeling tired or having little energy	0.784	0.178	1.07	0.96
5. Poor appetite or overeating	0.708	0.092	0.72	0.97
3. Trouble falling or staying asleep, or sleeping too much	0.679	0.136	0.94	1.04
1. Little interest or pleasure in doing things	0.671	0.279	0.63	0.92
7. Trouble concentrating on things, such as reading the newspaper or watching television	0.523	0.366	0.50	1.02
2. Feeling down, depressed, or hopeless	0.508	0.503	0.66	0.87
9. Thoughts that you would be better off dead or of hurting yourself in some way	0.027	0.813	0.15	0.56
6. Feeling bad about yourself, or that you are a failure or have let yourself or your family down	0.235	0.769	0.47	0.80
8. Moving or speaking so slowly that other people could have noticed? Or the opposite – being so fidgety or restless that you have been moving around a lot more than usual	0.460	0.611	0.45	0.82

a Global Scale. Alpha = 0.837; total mean = 5.6; total variance = 27.9 ± 5.3; Kaiser–Meyer–Olkin (sample adequacy) = 0.871; Bartlett’s sphericity test = 945.6, 21 df, *p* = 0.0001; Hotelling’s *t*-test = 383.8, *F* = 47.03, *p =* 0.001; total explained variance = 55.3%.

The GAD-7 showed excellent internal consistency (Cronbach’s *α* = 0.881). The KMO value was 0.900, and Bartlett’s test was significant (*p* < 0.001). EFA supported a unidimensional structure, with all 7 items loading onto a single factor with eigenvalues >1, explaining 58.5% of the variance. These results align with prior validation studies reporting a robust 1-factor model (20, 25). Item-level results are presented in Table 3.

**Table 3.** GAD-7 factor analysis in cancer patients^a^ Table 3 long description.

Items	Factor 1	Mean	SD
2. Not being able to stop or control worrying	0.833	0.75	0.94
3. Worrying too much about different things	0.798	0.81	0.99
1. Feeling nervous, anxious, or on edge	0.797	0.69	0.86
4. Trouble relaxing	0.787	0.61	0.88
7. Feeling afraid as if something awful might happen	0.716	0.60	0.87
5. Being so restless that it is hard to sit still	0.713	0.45	0.81
6. Becoming easily annoyed or irritable	0.698	0.77	0.91

a Global Scale. Alpha = 0.881; total mean = 4.7; total variance = 22.9 ± 4.8; Kaiser–Meyer–Olkin (sample adequacy) = 0.909; Bartlett’s sphericity test = 1101.96, 21 df, *p* = 0.000; Hotelling’s *t*-test = 89.10, *F* = 13.49, *p* = 0.001; total explained variance = 58.5 %.

**Figure 1.**
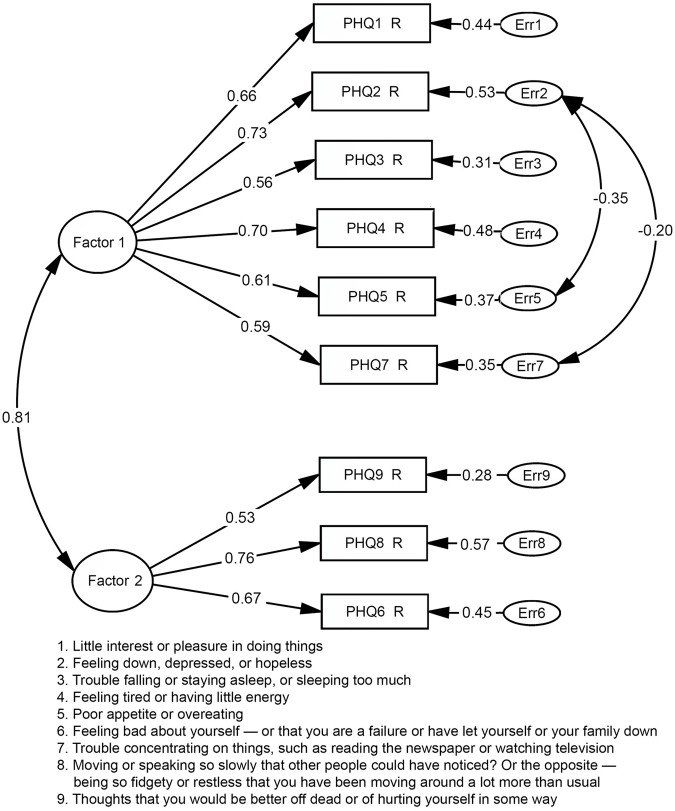
Two-factor CFA model for PHQ-9 in cancer patients. Chi square = 53.05, 24 df, *p* = 0.001, CMIN/df = 2.210; RMR = 0.028; GFI = 0.968, AGFI = 0.941. NFI = 0.945, CFI = 0.968, TLI = 0.953; RMSEA = 0.058 (0.037–0.080), Hoelter (sampling adequacy), *p* = 0.01, 289. Figure 1 long description.

**Figure 2.**
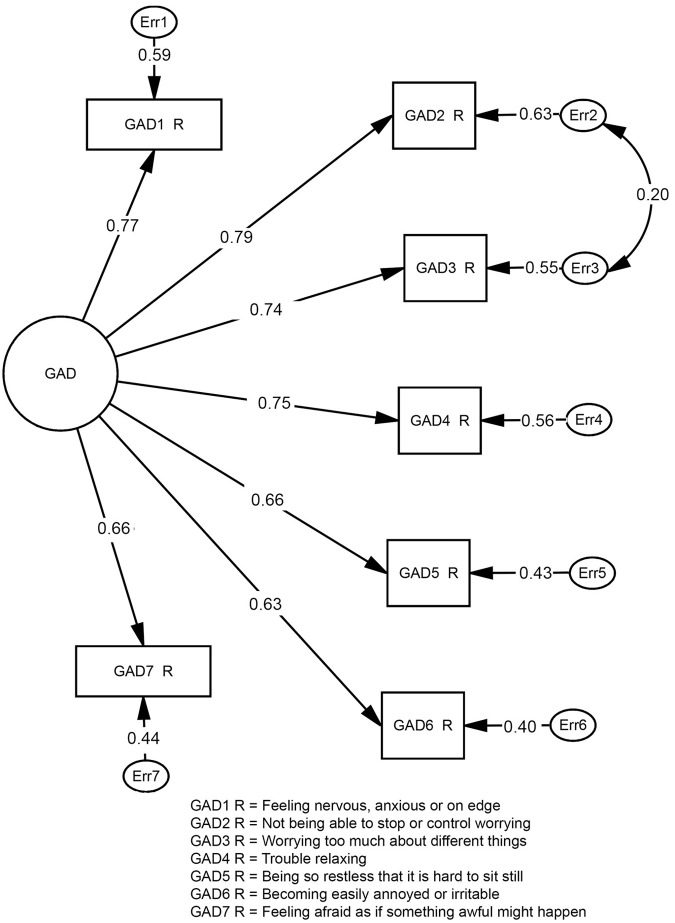
General single-factor CFA model for GAD-7 in cancer patients. Chi square = 21.69, 13 df, *p =* 0.060; CMIN/df = 1.668; RMR = 0.018; GFI = 0.982; AGFI = 0.962; NFI = 0.980; CFI = 0.992; TLI = 0.987; RMSEA = 0.043 (0.000–0.074); Hoelter (sampling adequacy), *p =* 0.05, 368. Figure 2 long description.

Figures 1 and 2 show the confirmatory factor analysis evaluating the fit of the 2-factor model for the PHQ-9 (Figure 1) and the single-factor model for the GAD-7 (Figure 2). In both models, we used the maximum likelihood method to estimate the data through the global fit indexes, the absolute value of χ2 (CMIN), and the ratio of χ2/degrees of freedom (CMIN/df), to confirm whether there were any errors in the variances and null covariances. The proposed changes were made in the modification indexes (Modification Indexes, MI). To improve the adjustment of the model, the AMOS® program version 23 was used.

## Discussion

The present study evaluated the psychometric properties of the PHQ-9 and GAD-7 in a sample of Mexican patients with cancer, providing evidence of their reliability, factorial validity, and clinical utility in this population. Both instruments demonstrated strong internal consistency and adequate model fit, supporting their use as brief screening tools for depressive and anxiety symptoms in oncology settings.

### Interpretation of findings

Consistent with prior research, the GAD-7 showed a robust unidimensional structure (Spitzer et al. 2006; Orozco et al. 2013), suggesting that anxiety symptoms in cancer patients cluster along a single continuum that integrates cognitive, emotional, and somatic manifestations. This pattern aligns with the multifactorial nature of cancer-related anxiety, which may arise from diagnosis, treatment demands, changes in body image, and disruptions in social and occupational functioning (Pitman et al. 2018; Semenenko et al. 2023). Given the convergence of these anxiety symptoms, a single-factor model appears clinically coherent and supports the use of the GAD-7 as a global indicator of anxiety severity.

In contrast, the PHQ-9 yielded a 2-factor structure, distinguishing somatic-affective symptoms from cognitive symptoms such as hopelessness and thoughts of death. This pattern has been reported in previous studies involving cancer patients and individuals with chronic medical conditions (Richardson and Richards 2008; Elhai et al. 2012; Petersen et al. 2015; Hinz et al. 2016). The differentiation between somatic and cognitive components is particularly relevant in oncology, where physical symptoms such as fatigue, appetite changes, and sleep disturbances may reflect both disease burden and depressive processes. The ambiguous loading of the psychomotor item (restlessness/slowed movements) has also been noted in other studies and may reflect treatment-related effects rather than pure depressive symptomatology. These findings underscore the importance of interpreting PHQ-9 scores within the clinical context of cancer care.

### Clinical implications

The use of brief, reliable instruments such as the PHQ-9 and GAD-7 can facilitate timely referral to psycho-oncology services and support stepped-care models. Routine screening using validated tools is recommended by international guidelines (British Coulumbia 2013; Joffres et al. 2013; Cleare et al. 2015; Bandelow et al. 2022) and is essential for identifying patients who may benefit from psychosocial interventions, particularly those with risk factors such as advanced disease, low social support, or a history of mental health problems (Linden et al. 2012; Ghanem et al. 2020; Shalata et al. 2024).

Evidence-based interventions, including cognitive behavioral therapy, mindfulness-based approaches, and meaning-centered psychotherapy, have demonstrated effectiveness in reducing symptoms of anxiety and depression and improving quality of life in cancer patients (Richardson and Richards 2008). Incorporating these tools into routine care may help reduce the treatment gap observed in many oncology settings, particularly in Latin American populations where stigma and limited access to mental health services remain significant barriers (Interian et al. 2010; Yanez et al. 2016).

### Relevance for the Mexican oncology context

The high prevalence of comorbid chronic conditions in Mexican cancer patients (Edwards et al. 2014; Sarfati et al. 2016) highlights the need for screening tools that can differentiate between disease-related symptoms and symptoms of anxiety and depression. The PHQ-9’s 2-factor structure may be particularly useful in this regard, allowing clinicians to interpret somatic symptoms with greater nuance. Additionally, national clinical practice guidelines emphasize the importance of standardized psychosocial assessment and referral pathways in oncology (Instituto Mexicano del Seguro Social 2017; Galindo-Vázquez and Costas-Muñiz 2019). The present findings provide empirical support for integrating the PHQ-9 and GAD-7 into these guidelines and into routine clinical workflows.

### Strengths and limitations

Strengths of this study include the use of culturally adapted versions of both instruments, a sample size adequate for factor analysis, and the application of both exploratory and confirmatory methods. However, several limitations should be noted. First, the study relied on self-report measures, and diagnostic interviews were not conducted; therefore, conclusions regarding formal psychiatric diagnoses should be drawn with caution. Second, test–retest reliability was not assessed.

Future research should examine the temporal stability, predictive validity, and sensitivity to change of both instruments, as well as potential variations in factorial structure across cancer types, treatment phases, levels of symptom burden, and sociodemographic groups. Longitudinal studies are also needed to clarify how depressive and anxiety symptoms evolve across the disease trajectory and how screening results may inform clinical decision-making. In addition, qualitative research may help elucidate cultural factors that shape symptom reporting, particularly among older adults who may underreport symptoms of anxiety and depression because of stigma or communication barriers (Interian et al. 2010; Yanez et al. 2016).

Overall, the present study provides robust evidence supporting the validity and reliability of the PHQ-9 and GAD-7 for assessing depressive and anxiety symptoms in Mexican patients with cancer. The PHQ-9 demonstrated a 2-factor structure that distinguished somatic-affective symptoms from cognitive symptoms, whereas the GAD-7 showed a stable unidimensional structure, consistent with findings from international validation studies (Spitzer et al. 2006; Richardson and Richards 2008; Elhai et al. 2012; Orozco et al. 2013; Petersen et al. 2015; Hinz et al. 2016). Taken together, these findings underscore the clinical relevance of both instruments for capturing the multidimensional nature of symptoms of anxiety and depression in oncology.

Given their brevity, ease of administration, and strong psychometric performance, the PHQ-9 and GAD-7 appear well suited for routine screening in oncology settings. Their incorporation into clinical workflows may facilitate the early identification of psychological symptoms, support timely referral to psycho-oncology services, and contribute to the evaluation of psychosocial interventions. This is particularly relevant in the Mexican context, where psychosocial needs in cancer care remain substantial and are often insufficiently addressed (Instituto Mexicano del Seguro Social 2017; Galindo-Vázquez and Costas-Muñiz 2019). Continued validation efforts may further strengthen the evidence base for implementing standardized psychosocial screening practices in oncology across Latin America.

## Table 1 Long description

The table summarizes sociodemographic and clinical characteristics for 357 cancer patients, reporting counts and percentages plus medians with 25th to 75th percentile ranges. Median age was 56 years (44 to 65). Sex distribution was 59% female (211) and 41% male (146). Most lived outside Mexico City (63%, 226) versus 37% in Mexico City (131). Marital status was most often married (46%, 165), followed by single (26%, 96), divorced (13%, 49), other (7%, 27), and widowed (5%, 20). The median number of children was 2 (1 to 3). Cancer stage was most commonly stage 4 (32%, 113), with stages 2 (25%, 90), 3 (22%, 76), and 1 (21%, 75) fairly similar. The most frequent cancer type was breast (34%, 120), followed by other types combined (14%, 50) and prostate (12%, 43), with several smaller groups such as head and neck and testicle (each 9%, 33). Comorbidities were present in 33% (118); specific comorbidities each affected about one in ten patients (hypertension 10%, diabetes 10%, other 10%), and 4% had both hypertension and diabetes. Functional status scores were generally high, with median Karnofsky 90 (90 to 100) and median ECOG 1 (0 to 1).

Navigate back to Table 1.

## Table 2 Long description

The table lists nine PHQ-9 symptom items for cancer patients with two factor loadings plus each item’s mean score and standard deviation. Factor 1 has the strongest loadings for fatigue or low energy (0.784), appetite change (0.708), sleep disturbance (0.679), and low interest or pleasure (0.671), suggesting a somatic and anhedonia cluster. Factor 2 is dominated by thoughts of death or self-harm (0.813) and feeling like a failure or letting others down (0.769), with psychomotor change also loading moderately (0.611). Depressed mood loads similarly on both factors (0.508 on Factor 1 and 0.503 on Factor 2), indicating it is not clearly specific to one factor. Mean symptom levels are highest for fatigue (mean 1.07) and sleep problems (0.94), and lowest for suicidal thoughts (0.15). Standard deviations range from 0.56 for suicidal thoughts to about 1.04 for sleep problems, showing more variability for several common symptoms. Factor loadings indicate association with each factor and do not by themselves prove distinct clinical categories or causation.

Navigate back to Table 2.

## Table 3 Long description

The table reports a one-factor solution for seven GAD-7 anxiety items in cancer patients, listing each item’s loading on Factor 1 plus its mean and standard deviation. Factor loadings are all high and tightly clustered, ranging from 0.698 to 0.833, suggesting the items reflect a common underlying anxiety dimension. The strongest loading is for “Not being able to stop or control worrying” at 0.833, followed by “Worrying too much about different things” at 0.798 and “Feeling nervous, anxious, or on edge” at 0.797. The weakest loading is “Becoming easily annoyed or irritable” at 0.698, with “Being so restless that it is hard to sit still” close at 0.713. Item means range from 0.45 for restlessness to 0.81 for worrying too much, with most other means between 0.60 and 0.77. Standard deviations range from 0.81 to 0.99, indicating similar variability across items. Because only one factor is shown, the table supports a single overall anxiety factor but does not compare alternative factor structures.

Navigate back to Table 3.

## Figure 1 Long description

The diagram illustrates a two-factor confirmatory factor analysis model for the PHQ-9, displaying factor loadings and error terms. At the center, two circles labeled Factor 1 and Factor 2 are connected by a line with a value of 0.81, indicating a correlation between the factors. Factor 1 is linked to seven rectangles labeled PHQ1 R through PHQ7 R, each with corresponding error terms labeled Err1 through Err7. The loadings from Factor 1 to these rectangles are 0.66, 0.73, 0.76, 0.70, 0.60, 0.70 and 0.60, respectively. The error terms have values of minus 0.44, minus 0.53, minus 0.31, minus 0.48, minus 0.35, minus 0.48 and minus 0.35. Factor 2 is connected to three rectangles labeled PHQ9 R, PHQ8 R and PHQ6 R, with error terms Err9, Err8 and Err6. The loadings from Factor 2 to these rectangles are 0.53, 0.76 and 0.67, respectively, with error terms of minus 0.28, minus 0.57 and minus 0.45. Additionally, there are curved lines connecting PHQ3 R to PHQ4 R and PHQ5 R to PHQ6 R, with values of minus 0.35 and minus 0.20, respectively. Below the diagram, a list of nine items describes symptoms related to depression, such as little interest or pleasure in doing things, feeling down or hopeless and trouble sleeping. These items correspond to the PHQ labels in the diagram.

Navigate back to Figure 1.

## Figure 2 Long description

The diagram illustrates a single-factor confirmatory factor analysis model for GAD-7. At the center is a circle labeled 'GAD', representing the general factor. Lines extend from this circle to seven rectangles labeled 'GAD1 R' through 'GAD7 R', each representing a specific item related to generalized anxiety disorder. The line connecting 'GAD' to 'GAD1 R' has a factor loading of 0.77 and an error term labeled 'Err1' with a value of 0.59 is shown above 'GAD1 R'. Similarly, 'GAD2 R' is connected with a factor loading of 0.74 and an error term 'Err2' with a value of 0.20 is depicted. 'GAD3 R' has a factor loading of 0.75, with an error term 'Err3' valued at 0.55. 'GAD4 R' is linked with a factor loading of 0.75 and an error term 'Err4' valued at 0.56. 'GAD5 R' shows a factor loading of 0.66, with an error term 'Err5' valued at 0.45. 'GAD6 R' has a factor loading of 0.63, with an error term 'Err6' valued at 0.40. Finally, 'GAD7 R' is connected with a factor loading of 0.44 and an error term 'Err7' valued at 0.66. Below the diagram, descriptions for each GAD item are provided: GAD1 R is feeling nervous, anxious, or on edge; GAD2 R is not being able to stop or control worrying; GAD3 R is worrying too much about different things; GAD4 R is trouble relaxing; GAD5 R is being so restless that it is hard to sit still; GAD6 R is becoming easily annoyed or irritable; GAD7 R is feeling afraid as if something awful might happen.

Navigate back to Figure 2.
